# Start moving - benefits of an onsite workplace health program in the age of digitalization

**DOI:** 10.1186/s12995-021-00338-8

**Published:** 2021-10-12

**Authors:** Borle Prem, Boerner-Zobel Franziska, Bias Harald, Voelter-Mahlknecht Susanne

**Affiliations:** Charité - Universitätsmedizin Berlin, corporate member of Freie Universität Berlin and Humboldt-Universität zu Berlin, Institute of Occupational Medicine, Augustenburger Platz 1, 13353 Berlin, Germany

**Keywords:** Physical activity, Coaching, Multilevel approach, Self-efficacy, Workplace

## Abstract

**Background:**

The process of digitization should simplify our work and improve related processes (i.e. quality, transparency). Moreover, it enables the home office, which is greatly expanded due to the current pandemic. Regarding workplace health, it should be noted that with increasing digitization, physical activity decreases, and as a result, the number of work-related diseases will increase. On the other hand, increasing digitization also offers promising opportunities for new approaches to workplace health promotion. With these positive as well as negative effects in mind, we designed a workshop to increase physical activity at work. This protocol describes our approach to a live workshop concept.

**Methods:**

We use a randomized controlled trial with two intervention groups: a live workshop with and without additional reminders. The workshop intervention design consists of a baseline measurement, two workshops, and one follow-up measurement. Each workshop takes place in small groups (*n* < 11). We use a randomized allocation to both groups. To control for health-related effects and the expected behavior change we examine (i) physical activity (i.e. active time, taken steps, etc.) by a tracking device (ii) physical wellbeing, motivation, and volition by an online questionnaire, and (iii) participants also report physical activity by a diary. All measurements are taken one week before the respective workshop and 24 weeks after the initial baseline measures.

**Discussion:**

A live workshop offers advantages such as very personal interactions and a low technical effort. However, during the current pandemic, there are some limitations (i.e. small groups, pay attention to hygienic guidelines). Based on the upcoming experiences of this workshop, a web-based approach might offer some advantages (i.e. easier daily implementation, independent from a participant’s location) regarding home office workplaces and the increasing digitization. On the other hand, there are also mandatory requirements as a stable internet connection and technical equipment (i.e. webcam, microphone). Overall, a step by step development of a web-based workshop, based on the experience of the live workshop, can be regarded as advantageous.

**Trial registration:**

Since this article reports a health promotion intervention concept with human participants, we registered it in the German Clinical Trials Register (DRKS). Number:DRKS00021512, Date:30.10.2020.

## Introduction

Today, digitized office workplaces are equipped with a computer, printer, telephone, webcam, and in the best case height-adjustable desks to meet ergonomic requirements. The latter also enables office workers to switch their position between sitting and standing, to trigger some physical activity. However, most office and home-office workplaces still have no height-adjustable desk and office workers spend most of their working hours sitting (i.e. while working on the computer, attending meetings or conferences, or making telephone calls) [1]. Additionally, during the after-work hours, people relaxing on the couch, in front of the TV, and use the benefits of a smart home, which in turn increases the sedentary behavior [1]. Regarding the described behavior, it is obvious that the process of digitization might be also accompanied by negative effects (i.e. reduced physical activity and mental stress) [1, 2]. Especially, since the current pandemic is causing a rapid development of digitization, it can be feared that inactive behavior increases.

Importantly, inactivity increases the risk of lifestyle diseases like cardiovascular events, type II diabetes, or cancer [3]. For example, increased daily sitting times (i.e. six to eight hours in total) are associated with a higher risk of mortality [4, 5]. Additionally, it is accepted that long periods of sitting or inactivity are associated with musculoskeletal disorders (MSD, i.e. back pain). MSD was the most frequent reason for days of incapacity to work in 2014 [6] and in the current absence from work report [7], MSD are still the second most frequent reason.

One interesting approach to countermeasure inactivity is a twofold strategy of reducing sitting time and increasing physical activity [8]. The first one is strongly influenced by habits [9], thus interventions should consider approaches for behavioral change. The second part of the strategy is as well important, as the negative effects of long periods of sitting can be positively influenced by increased physical activity [3, 10]. Moreover, a reduced sitting time without physical activity shows only minor effects [11].

A recent review indicated that exercise programs, workplace support, participative approaches, or cognitive-behavioral programs are beneficial [12]. In this vein, multi-component programs, which include the individual, the organization, and the workplace, should be preferred [12–15]. For example, [15] reported three multi-component interventions in which height-adjustable desks combined with behavioral interventions (e.g. goal-setting, self-monitoring, problem-solving, etc.) were used. In this review, the authors showed that multi-component interventions and single workplace-related interventions (e.g. height-adjustable desks) are most effective in reducing sitting time. For multi-component approaches, the literature showed moderate but heterogeneous evidence regarding the reduction of sitting times at the workplace [16, 17]. Despite these positive results, more research is needed, as the number of multi-component studies compared to single intervention studies is still too small [13, 15, 17].

Another review that focused on a reduction of sitting time described approaches and techniques, based on the Behavior Change Wheel [18], and named four principal intervention approaches: (i) knowledge transfer, (ii) efforts at persuasion, (iii) environmental restructuring, and (iv) training [19]. The authors also described other behavioral change techniques (i.e. problem-solving, goal-setting, self-monitoring, social support, feedback), which have also been promising. Furthermore, other researchers reported that self-efficacy and action planning can influence the implementation of physical activity positively [20]. Self-efficacy is defined as a person’s beliefs that events in their own life can be influenced through their abilities [21, 22]. Regarding the occupational context, self-efficacy can be subdivided into (i) occupational, (ii) social, (iii) self-oriented emotional, and (iv) other-oriented emotional [23]. Additionally, the authors refer to some studies that describe positive associations between self-efficacy and healthy work. Action planning is one of the different techniques towards a behavioral change [19] and includes the “when”, “where” and “how” of planned behavior [20]. These theoretical constructs are quite useful, since a self-efficacy questionnaire, for example, can also provide information about the effectiveness of action plans [24]. However, the constructs are also discussed critically. Studies have shown that only action planning has direct effects on physical activity whereas self-efficacy or other social-cognitive factors have indirect [24, 25] or no effects [26, 27]. Our approach refers to both the above-mentioned principles for a behavioral change as well as a social-cognitive process model (i.e. Health Action Process Approach, HAPA [28, 29]) that includes self-efficacy and action planning. The latter is described more detailed in the methods section.

A further aspect of promising workplace interventions is the usage of reminders (i.e. advertising or describing additional content, offering assistance, or reminding workshop content) to stabilize the favored behavior. Previous studies showed that text messages (e.g. per short message service, SMS) have short term effects regarding strategies of disease prevention and management (e.g. healthier diet, increased physical activity) [30] or achieving behavior change goals (e.g. eating more vegetables, increased walking time per day) [31]. In contrast, a review of cue-reminders (i.e. objects that were introduced during the intervention and should remind a target behavior) showed insufficient evidence [32]. Many of those studies differed regarding the frequency of reminders, but some results indicate that: (i) frequent reminders are more effective [33], (ii) there should be a personal contact [33], and (iii) technology-based strategies (e.g. Email, SMS) seem to have small-to-moderate positive effects on engagement compared to no strategy [34]. Thus, reminders seem to be advantageous for behavior change interventions.

As an intervention, we planned a group-based participatory workshop including a knowledge transfer of health behavior at work, an interactive discussion about how to deal with opportunities and barriers of increased physical activity practice sessions, and action planning regarding increased physical activity and reducing sitting times at the workplace. The HAPA postulates that health behavior change requires both a motivational and a volitional phase. After forming the intention to engage in a certain health behavior in the motivational phase, self-regulatory strategies such as planning are essential for behavioral enactment [29]. During the workshop participants are supported in formulating action plans, that help to bridge the *intention-behavior gap* [35] and might facilitate engagement in physical activity and reduction of sitting times at the workplace. Results of a current systematic review indicated that physical activity is in turn associated with increased well-being [36].

The participation should motivate and offer new perspectives of increasing physical activity at the workplace. We have also designed the workshop in such a way that our concept can be used in various companies. In this first step, we want to prove our concept in medium-sized organizations. Due to the current pandemic situation, there is an increasing number of people working in separate offices without any contact with other co-workers. To ensure that these changes do not lead to increased inactivity, new work-related health offers or activity programs should be established. We developed a workshop which takes all the above-mentioned points into account. The use of computer prompts combined with providing information reduces sitting time on average by 14 to 96 min per day*.* In this early stage, we want to prove the concept of our live approach in small groups and hypothesize that the participants show positive health effects (i.e. well-being, decreased MSD) and positive adaptations in their health behavior (i.e. increased physical activity). We hypothesize that additional reminders improve health-related effects. In addition, we want to develop our workshop design in the framework of eHealth applications or a web-based solution, since the integration of such approaches might improve the efficiency of health programs at work [37].

## Methods

### Participants

We recruit office desk workers aged between 18 and 67 years from different organizations (i.e. public administration with standard office workplaces) in Germany. Two intervention lines are planned: one live workshop group with and one without reminders. After attending the first workshop participants in the appropriate intervention group will receive reminders via e-mail twice a week. The e-mails remind the participants of the possibilities to be physical active at the work place previously introduced through the workshop. Overall, eight e-mails will be sent until participants answer the second online survey one month after participating in the workshop. We use a randomized allocation of the participants to both groups. As inclusion criteria, we define an age group between 18 and 67 years and the employee council of each institution has to approve the participation. Additionally, participants are asked not to take part in other studies during the time of the workshop intervention. Participants are excluded if they take part in only one workshop or do not fill in the questionnaires (missing answers > 30%) appropriately. All participants will be informed about the content and objectives of our study, they can ask questions, and they can withdraw from the participation at any time. As a prerequisite for participation, participants of our study sign a declaration of consent. A meta-analysis [38] revealed small intervention effects of workplace physical activity interventions (d = 0.21). Based on this assumption, power-analysis (G-Power 3.1) indicated in sum 168 participants for an ANOVA with repeated measures (within-between interaction). Our calculation included *f* = 0.11, *α* = 0.05, β = .80, three groups, and three measurements.

### Study-aim and procedure

Our study aims to examine health-related effects from two slightly different live workshops with office workers. Therefore, we contact health counselors at various companies in Germany. If they are interested in participating in our study, further details will be discussed and the employee council and management level will be involved. If the management approves our study, the health counselors will be interviewed and will help to distribute the information among the employees (i.e. via email). In an email, participation in one of our groups will be clearly explained. The study information and the consent form are attached to this email, with additional contact details of the study supervisors (i.e. regarding upcoming questions). As a prerequisite for participation, these documents must be signed and returned. The participants have the opportunity to choose a suitable date from a list of workshop dates in their company. Each participant receives a link to participate in the online questionnaire as well as an activity diary one week before their first workshop. Additionally, we use accelerometers to objectively measure physical activity during this week. Accelerometers with instructions, activity diaries and an online survey will be sent to all consenting participants for data collection. The instructions explain how to attach the accelerometer to the participant’s thigh and hip. Previous studies have indicated three days are sufficient to observe a stable pattern of physical activity [39]. Not wearing the accelerometer for 3 successive days leads to exclusion of the participant from the study.

**Fig. 1:**
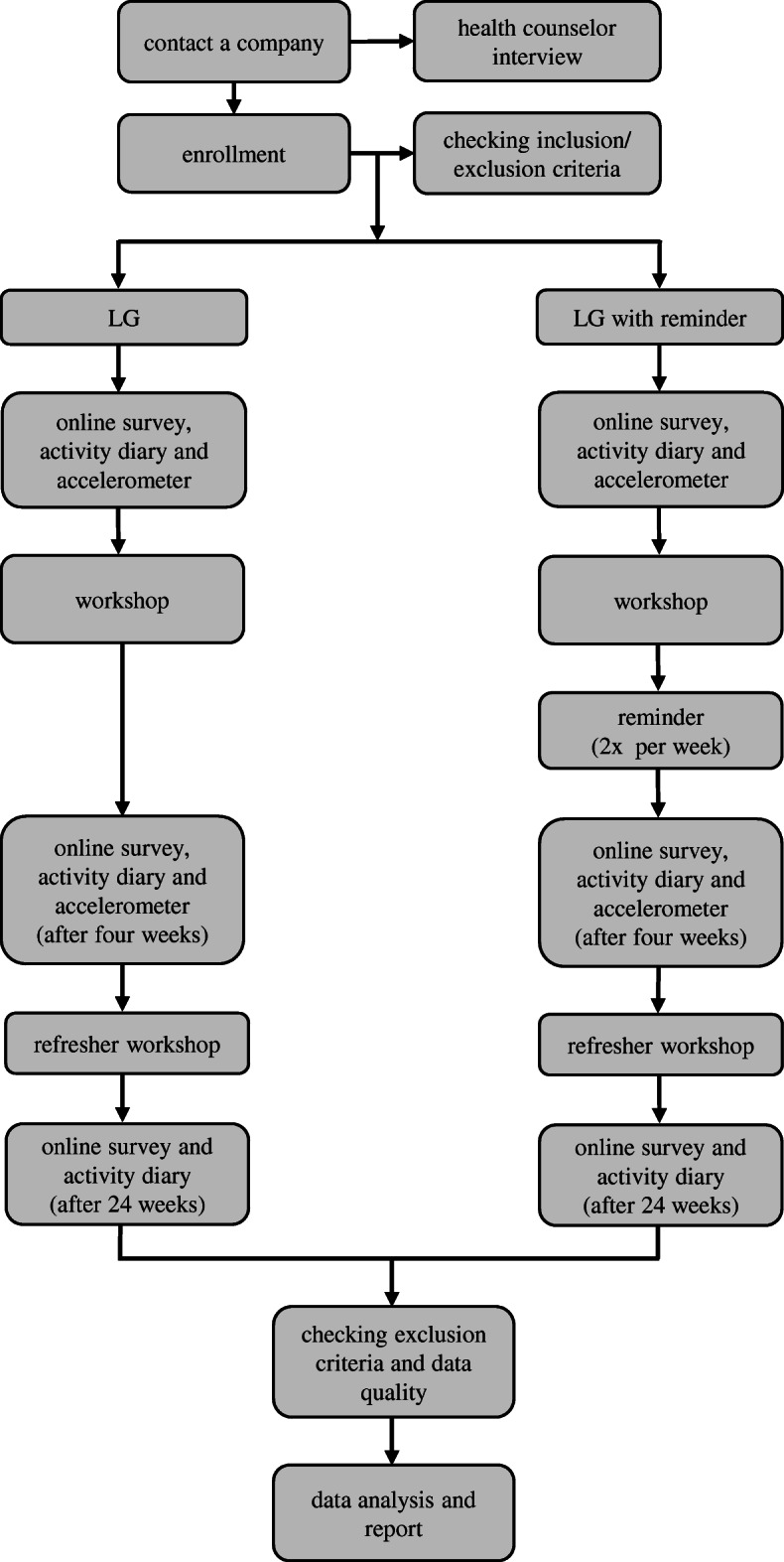
Flowchart of the study protocol from contact companies to data analysis (LG = live workshop group). Participants receive a reminder via email

This procedure is repeated one week before the refresher workshop and after six months. We have no blinding of our workshop supervisors and outcome assessors or data analysts. In each group data collection starts one week before the first workshop. The study procedure is presented in Fig. 1.

The study lasts about six months for each group. Each workshop and measurement for data collection is promoted by the institutions’ health counselor. Our workshop address three important components for office workers’ health promotion: organizational (i.e. structures, health services), workplace (i.e. equipment, premises), and individual (i.e. health behavior, motivation) components. Details of our intervention are described below. Besides the two interventions groups participants of the control group will receive the pre- and post-study questionnaire.

### Intervention

The first workshop lasts about two hours, the refresher workshop only one hour. Each workshop consists of active and passive parts. Table 1 describes the first workshop including active and passive parts. The refresher workshop includes some new activities (see part III in Table 1) as well as an interactive question-answer part. In the latter participants can ask questions about the workshop and further individual strategies, and can discuss adaptations. This concept is used in a live workshop with and without reminders. The reminders are used to retain the acquired contents and knowledge of the first workshop and to give incentives for physical activity.

**Table 1:** Description of main contents during the workshop; participants were actively (i.e. physically active) or passively involved

***Part***	***Content***	***Participants’ involvement***
I	Workshop trainers give some general information about inactive lifestyle and behavior (i.e. statistics) at German workplaces and resulting health-related issues (i.e. physically, social, psychological), based on recent studies. Different concepts for increased physical activities at the workplace are shown and participants are asked in anonymized live-questions about their daily physical activity.	passive
II	The trainers give instructions on adequate health safety standards regarding the office desk setting, based on German laws and regulations. This includes the rights & duties as well as the health-related behavior of office workers at their workplace (i.e. position of the desk, equipment, sitting/standing positions) in Germany. Again, the participants are asked for their participation in anonymized live-questions about these topics.	passive
III	Trainers show some exercises from the areas of mobilization, strengthening, and stretching. All examples can be performed at the workplace. At the end of this tryout, some recommendations for physical activity (i.e. how long and often exercises should be performed) are given.	active
IV	Trainers and participants of the workshop discuss opportunities and barriers to daily physical activity in individual group activities. Experiences are shared and the group works on the conception of action plans. Finally, each participant should create his/her plan.	active

The live workshops take place according to the current hygiene guidelines (i.e. visit https://www.infektionsschutz.de/coronavirus/). These guidelines currently recommend a minimum interpersonal distance of 1.5 m, wearing a personal protective equipment such as a face mask, and good hygiene practices including hand washing etc., (including hand washing for at least 20 s, avoiding touching each other, sneezing into the elbow, etc.). Moreover, further precautionary arrangements are made, for example, smaller size groups, large rooms for sufficient interpersonal distance, regular airing, and disinfection of all tools that are used.

### Measurements

In the first step, we conduct a structured telephone interview with the health counselor from the company. The respective manual will be attached as supplementary material (S1). The aim is, to explore health offerings, courses, and general attitudes towards employees’ health, health promotion, and programs. Subsequently, small discussion parts of our workshop will be adapted to the company’s framework (i.e. if offices have no height-adjustable desks, we won’t discuss it). Using this approach, we create a crucial prerequisite for multilevel interventions at the workplace: the inclusion of the organization [13].

The subsequently described questionnaires (i.e. Nordic Musculoskeletal Questionnaire, Questionnaire of physical well-being, and Health Action Process Approach) are embedded in one online questionnaire that was built using LimeSurvey, an open-source online survey tool. In compliance with the “*Datenschutz Grundverordnung*” (DSGVO), we hosted the survey on an internal server from the Charité – Universitätsmedizin Berlin, and participants get an email-link for participation.

#### Nordic musculoskeletal questionnaire (NMQ)

This questionnaire aims to detect musculoskeletal stress in different regions of the body [40]. The NMQ consists of two parts: first, general information about the participant including the individual situation at work, and second, detailed questions about previous and existing physical stress. The latter also examines special regions of the body (i.e. neck, shoulder, lower back). All questions include the duration and frequency of physical stress, especially during the last 12 months and the last seven days. Previous research concluded that the NMQ is sensitive, repeatable, and a useful screening tool [41]. We use this questionnaire in two ways: (i) as a screening tool to adapt the physical activities in our refresher-workshop, and (ii) to record changes in physical stress a few weeks after the workshop.

#### Questionnaire of physical well-being (FEW-16)

The FEW-16 contains some statements about the body, physical capacity, and relaxing. It is a retrospective assessment of the general situation during the last three weeks. Therefore, participants have to indicate the extent to which they agree with the individual statements. The questionnaire contains four scales each includes four items (i.e. stress resistance, ability to enjoy, vitality, and inner peace). Each item is assessed by a six-point Likert scale with a range from zero: “not applicable at all” to five: “fully applicable”. Kolip and Schmidt (1999) reported high internal consistencies (i.e. α = .82 to .92) for the total scale and the subscales and validated the questionnaire with questionnaires on quality of life and functional impairments [42]. Although the FEW-16 contains four scales, in a non-clinical context the physical well-being seems to be one-dimensional [43]. Thus, in our study, we will use the sum-score of all sub-scales. The score ranges from zero to 80 points, in which higher scores represent higher well-being.

#### Health action process approach (HAPA)

**Fig. 2:**
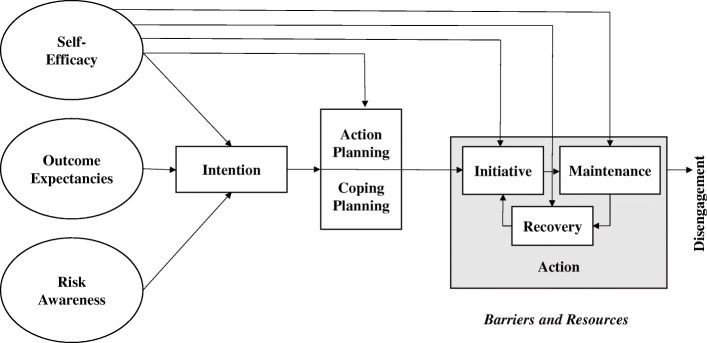
HAPA-model by Schwarzer R. (adapted from Schwarzer, 2008)

The HAPA model (Fig. 2) assumes health behavior as a process with at least two stages: motivation and volition [28, 29]. The first stage includes individuals’ risk perception, outcome expectancies, and task self-efficacy, and results in a formed intention. The latter stage includes planning, execution, and maintenance of health behavior, and leads to actual behavior change. The HAPA model was proven in some empirical studies [29]. The author reported that especially the improvement of action planning and coping planning in patients motivated to increase their physical activity. The questionnaire as well as further information can be received from *http://userpage.fu-berlin.de/%7Ehealth/hapa.htm*.

The questionnaire contains different subscales (i.e. self-efficacy, planning, intention, outcome expectations, and risk perception). Subscale items are assessed by a four-point Likert scale (i.e. from zero = “not applicable at all” to three: “fully applicable”), except the risk perception with a five-point Likert scale. Scores are computed by adding or averaging the answers over all items.

#### Physical activity diary (PAD)

The physical activity diary is adapted to the German-PAQ-50+ [44], which is a retrospective assessment of one month of daily physical activity. Huy and Schneider (2008) reported acceptable reliability but did not validate the questionnaire as the PAQ-50+ consists of two already validated questionnaires (i.e. YPAS and PASE) [40]. We use the PAQ-50+ in our physical activity diary to assess daily physical activity. Answers are given in hours/week and are added to a sum-score that is multiplied by an appropriate energy score (i.e. MET-value).

#### Activity tracker

Physical activity is objectively measured by ActivPAL™ (PAL Technologies), which is a validated and reliable accelerometer for activities like sitting, standing, or walking [45, 46]. Especially in sedentary behavior, the ActivPAL™ is a precise and sensitive measurement tool [47]. The sensor is attached to the right thigh and measures not only the number of steps but also various activity patterns. In this study, the measurement is performed over seven consecutive days to examine the average physical activity and to have a comparative value to the PAD.

### Statistics

All data are processed to enhance quality; for example, in case of missing data, we conduct a missing value analysis (MVA) with a subsequent imputation procedure. Outliers are checked manually. Then, it is planned to perform analyses for repeated measurements of physical wellbeing, HAPA subscales, and the results from the physical activity diary. Results from the Nordic Musculoskeletal Questionnaire are analyzed descriptively. For all analyses, the significance level is set to 0.05.

### Monitoring and ethics

There will be no data monitoring committee. However, data will be regularly monitored (i.e. following each workshop) by the principal investigators. There is no external funding for this study and therefore the principal investigators have no financial or other competing interests. Adverse events during the workshops or within data collection will be logged and reported. The study is approved by the ethics committee of Charité Universitätsmedizin Berlin (No.: EA2/185/19). We have to point out that our ethics approval does not yet cover the implementation of reminders. This aspect is currently being submitted in a supplementary application. We assume that this aspect is ethically acceptable and that a corresponding positive vote can be handed in later. Other protocol amendments are not planned. In case an adjustment to the protocol is necessary, this will be indicated in the subsequent report of our results. By publishing this study protocol in advance as well as by using a study registration, all subsequent changes are traceable. Only the principal investigators of this study will have access to the final datasets. All data will be encoded and stored electronically on a password-protected external drive. All identifiable data (i.e. completed questionnaires) are secured as a single version in lockable cabinets that are in lockable rooms.

Only analyses based on aggregated data (*n* > 15) will be released back to the organization to ensure the anonymity of participants. The invitation to participate in the study will state that participation is voluntary and no negative consequences will impact those that choose not to participate. The overall study results and circumstances will be made available to all individuals irrespective of their group allocation.

## Discussion

We introduced a workshop to increase health-seeking behaviors in office workplaces in Germany. Our workshop is based on a combination of physical activity and health behavior planning.

At first, we want to indicate one minor limitation regarding our data acquisition: we have to validate our PAD. The diary is adapted to a validated questionnaire; however, that questionnaire has not been validated for daily usage. Moreover, the literature showed differences between self-reported (i.e. by questionnaires) and accelerometer-measured physical activity [48, 49]. Therefore, we use accelerometer-measured physical activity to prove our PAD in a sub-sample of our participants. If the result of our analysis shows insufficient validity, only the data from the accelerometry will be used. Another limitation refers to the recruitment process. The participants are recruited in different organizations and therefore have different pre-conditions (i.e. workplace equipment, health services) as well as differently effective health counsellors. Thus, we will collect data on the organization to control for potential experimenter effects between organizations.

In addition, there is some risk of inter-subject group bias if individuals in one group inform a member of the other group about reminders. In the follow-up survey, we will ask the group without reminders whether they received information about the procedure in the other group. We can then perform analyses according to whether participants received information about the other group and exclude them from the analyses, where necessary. Participants of the workshops might be less active in winter. Hence, the season could affect the amount of physical activity. As data of both intervention groups will be collected in the same season, they remain comparable. All these issues could affect our results and should be critically reviewed later.

Regarding the HAPA model, participants have different stages: motivational as well as volitional [28, 29]. Participants at the motivational stage first have to learn the physical activity is beneficial as it is associated with numerous positive effects. At the volitional stage, participants rather need help with the implementation in their daily routines. Our intervention targets both stages: (i) the motivational, as the workshop contains knowledge transfer about positive effects and ways to overcome barriers of physical activity, and (ii) the volitional, as the main contents are the performance of the physical activity and the learning of implementation strategies. A similar study on sedentary behavior used a personalized intervention approach to reduce such behavior [50]. The study failed regarding the sum of sedentary minutes per day but decreased the number of long periods of sedentary behavior. In contrast, another study showed that a multi-component intervention can change sitting times in favor of standing times [14]. As mentioned in the introduction, we use these and other described findings from the literature in our approach to developing optimal support for small and medium-sized companies in Germany.

We also have to look at our workshop critically because of the current pandemic. Currently, in Germany, it is only allowed to meet in small groups concerning the hygienic guidelines, which can limit our onsite workshop. Therefore, other options have to be discussed, for example, a web-based solution with an interactive video function. Such an adaptation is conceivable since our concept and the appropriate content can be easily transferred. A web-based workshop has also other advantages: (i) it is easier to implement in a daily routine, (ii) it is independent of the participants’ location, (iii) the capacity limits depend on a stable internet connection, and (iv) participants learn a web-based interaction that might be beneficial for other work processes. Thus, using a web-based solution as an eHealth tool is advantageous, however, there should be clearly defined rules (i.e. regarding data privacy and data security) for implementation [37]. Besides those rules, a recent study in German and Austrian leaders indicated that eHealth tools should include feedback from experts to increase the acceptance rates of a tool [51]. Another aspect regarding a web-based solution are the insufficient health promotion offers in small- and medium-sized companies in Germany, especially as these companies account for 90% of the entire sector [52, 53]. Web-based services are highly effective in terms of time and costs and could therefore support the implementation of health promotion offers in these companies. Considering all the approaches, our current study could be the first step towards a web-based workshop.

Summing up, our study includes a live workshop based on a multi-component approach that is promising to help increase physical activity in office workers. After data evaluation, we will review our hypotheses and results in line with the mentioned limitations critically. Thereby, we will be able to develop an effective workshop for health promotion that could be implemented at home and office workplaces. Especially a web-based solution, with its potential to reach many people in an increasingly digitalized work environment, will be developed and tested based on our first results.

Possible implementation areas would be the classic workplace and new forms of work, for example, home-office and mobile working.

## Data Availability

Currently, there is no data available, as the study has not yet started.
