# Correction to: Start moving - benefits of an onsite workplace health program in the age of digitalization

**DOI:** 10.1186/s12995-021-00340-0

**Published:** 2021-10-26

**Authors:** Prem Borle, Franziska Boerner-Zobel, Harald Bias, Susanne Voelter-Mahlknecht

**Affiliations:** Charité - Universitätsmedizin Berlin, corporate member of Freie Universität, Berlin and Humboldt-Universität zu Berlin, Institute of Occupational Medicine, Augustenburger Platz 1, 13353 Berlin, Germany

**Correction to: J Occup Med Toxicol 16, 46 (2021)**

**https://doi.org/10.1186/s12995-021-00338-8**

Following the publication of the original article [1], we were notified that the authors’ first and last names had been mistakenly swapped.

- Originally published names:
  Borle Prem, Boerner-Zobel Franziska, Bias Harald and Voelter-Mahlknecht Susanne
- Corrected names:
  Prem Borle, Franziska Boerner-Zobel, Harald Bias and Susanne Voelter-Mahlknecht

The original article has been corrected.
